# Effects of long-term weekly iron and folic acid supplementation on lower genital tract infection – a double blind, randomised controlled trial in Burkina Faso

**DOI:** 10.1186/s12916-017-0967-5

**Published:** 2017-11-23

**Authors:** Loretta Brabin, Stephen A. Roberts, Sabine Gies, Andrew Nelson, Salou Diallo, Christopher J. Stewart, Adama Kazienga, Julia Birtles, Sayouba Ouedraogo, Yves Claeys, Halidou Tinto, Umberto d’Alessandro, E. Brian Faragher, Bernard Brabin

**Affiliations:** 1 Division of Cancer Sciences, Faculty of Biology, Medicine and Health, School of Medical Sciences, University of Manchester, Manchester Academic Health Science Centre, Manchester, UK; 2 Centre for Biostatistics, Division of Population Health, Health Services Research and Primary Care, Faculty of Biology, Medicine and Health, School of Medical Sciences, University of Manchester, Manchester Academic Health Science Centre, Manchester, UK; 3 Department of Biomedical Sciences, Prince Leopold Institute of Tropical Medicine, Antwerp, Belgium; 4 Medical Mission Institute, Würzburg, Germany; 5 Faculty of Health and Life Sciences, Northumberland Building, University of Northumbria, Newcastle-upon-Tyne, England; 6 Clinical Research Unit, Institute for Research in Health Sciences, (IRSS-URCN), Nanoro, Burkina Faso; 7 Molecular Virology and Microbiology, Baylor College of Medicine, Houston, Texas USA; 8 Central Manchester University Hospitals NHS Foundation Trust, Microbiology Department, Manchester, UK; 9 Clinical Trials Unit, Prince Leopold Institute of Tropical Medicine, Antwerp, Belgium; 10 Medical Research Council Unit (MRC), Banjul, Gambia; 11 London School of Hygiene and Tropical Medicine, London, UK; 12 Clinical Division, Liverpool School of Tropical Medicine, Liverpool, UK; 13 Liverpool School of Tropical Medicine and Institute of Infection and Global Health, University of Liverpool, Liverpool, UK; 14 Global Child Health Group, Academic Medical Centre, University of Amsterdam, Amsterdam, The Netherlands; 15 Division of Cancer Sciences, 5th (Research) floor, St Mary’s Hospital, Oxford Road, Manchester, M13 9WL UK

**Keywords:** Lower genital tract infection, Iron, Antibiotics, Adolescents, Burkina Faso

## Abstract

**Background:**

Provision of routine iron supplements to prevent anaemia could increase the risk for lower genital tract infections as virulence of some pathogens depends on iron availability. This trial in Burkina Faso assessed whether weekly periconceptional iron supplementation increased the risk of lower genital tract infection in young non-pregnant and pregnant women.

**Methods:**

Genital tract infections were assessed within a double blind, controlled, non-inferiority trial of malaria risk among nulliparous women, randomised to receive either iron and folic acid or folic acid alone, weekly, under direct observation for 18 months. Women conceiving during this period entered the pregnancy cohort. End assessment (FIN) for women remaining non-pregnant was at 18 months. For the pregnancy cohort, end assessment was at the first scheduled antenatal visit (ANC1). Infection markers included Nugent scores for abnormal flora and bacterial vaginosis (BV), *T. vaginalis* PCR, vaginal microbiota, reported signs and symptoms, and antibiotic and anti-fungal prescriptions. Iron biomarkers were assessed at baseline, FIN and ANC1. Analysis compared outcomes by intention to treat and in iron replete/deficient categories.

**Results:**

A total of 1954 women (mean 16.8 years) were followed and 478 (24.5%) became pregnant. Median supplement adherence was 79% (IQR 59–90%). Baseline BV prevalence was 12.3%. At FIN and ANC1 prevalence was 12.8% and 7.0%, respectively (*P* < 0.011). *T. vaginalis* prevalence was 4.9% at FIN and 12.9% at ANC1 (*P* < 0.001). BV and *T. vaginalis* prevalence and microbiota profiles did not differ at trial end-points. Iron-supplemented non-pregnant women received more antibiotic treatments for non-genital infections (*P* = 0.014; mainly gastrointestinal infections (*P* = 0.005), anti-fungal treatments for genital infections (*P* = 0.014) and analgesics (*P* = 0.008). Weekly iron did not significantly reduce iron deficiency prevalence. At baseline, iron-deficient women were more likely to have normal vaginal flora (*P* = 0.016).

**Conclusions:**

Periconceptional weekly iron supplementation of young women did not increase the risk of lower genital tract infections but did increase general morbidity in the non-pregnant cohort. Unabsorbed gut iron due to malaria could induce enteric infections, accounting for the increased administration of antibiotics and antifungals in the iron-supplemented arm. This finding reinforces concerns about routine iron supplementation in highly malarious areas.

**Trial registration:**

Trial registration number NCT01210040. Registered with Clinicaltrials.gov on 27 September 2010

**Electronic supplementary material:**

The online version of this article (doi:10.1186/s12916-017-0967-5) contains supplementary material, which is available to authorized users.

## Background

Most genital tract commensals and pathogens require iron for growth and adhesion, including *Gardnerella vaginalis* [1], *Trichomonas vaginalis* [2] and *Candida albicans* [3]. In contrast, most *Lactobacillus* species, which maintain a healthy vaginal ecosystem, do not require an iron substrate [4]. There have been no previous randomised trials assessing whether vaginal infections are influenced by host iron status. Local iron is available from secreted lactoferrin [5], transudated ferritin and transferrin [6, 7], and menstrual haem iron. Host iron status could affect growth and adhesion of bacterial vaginosis (BV) [8], which is associated with a massive overgrowth of vaginal organisms. Dysbiosis enhances human immunodeficiency viral infectivity [9] and increases by two-fold the risk of preterm birth and perinatal death [10]. Higher concentrations of transferrin in cervico-vaginal fluid were correlated with preterm birth risk in a previous study, suggesting that iron availability must be considered [11].

In areas where anaemia is common, the World Health Organization (WHO) recommends routine iron supplementation [12]. It is currently unknown whether this affects bacterial profiles in the reproductive tract, especially in already iron replete women. In routine programmes, these women receive additional supplements despite there being good evidence that elevated iron stores predispose to systemic infection and inflammation [13]. However, data are absent regarding non-malarial infection risk following iron supplementation during pregnancy [14], or following intermittent supplementation in menstruating women [15]. In the context of a double blind, randomised, controlled, non-inferiority trial to assess safety of periconceptional iron supplementation in relation to malaria among young women in Burkina Faso [16], we assessed whether weekly iron supplementation, or baseline host iron status, affected markers of genital infection. The trial design provided two cohorts, namely a large nulliparous cohort who remained non-pregnant and received up to 18 months supplementation, and a primigravid cohort who conceived during the trial.

## Methods

The trial protocol and amendments were approved by ethical review boards and regulatory authorities at each collaborating centre. This sub-study was conducted within a randomised trial of the safety of weekly iron and folic acid supplementation in young women exposed to malaria. The primary specified outcome was BV prevalence, whereas the secondary outcomes were non-BV Nugent scores, vaginal discharge and pH > 4.5. Finally, exploratory outcomes were microbiota and drug prescriptions (antibiotics, antifungals, analgesics). Additional data on the main trial and other details relevant to this paper are provided in Additional files 1, 2, 3 and 4.

### General procedures

Between April 2011 and January 2014 a randomised, double blind, controlled trial was conducted in rural Burkina Faso in the Nanoro Health and Demographic Surveillance System area [17], situated 85 km from Ouagadougou (Additional file 1). Two previous surveys in Burkina Faso indicated a BV prevalence of approximately 6–8% [18, 19]. HIV prevalence is low and reported as 1.2% among women aged 15–49 years and 0.76% among pregnant women [20]. Women were recruited from 30 villages and individual and guardian written consents were obtained. Healthy nulliparous, non-pregnant women aged 15–24 years received either weekly ferrous gluconate and folic acid (intervention), or folic acid alone (control) as a directly observed therapy. Participation continued for 18 months for women who did not conceive or, if they became pregnant, they entered the pregnant cohort. Weekly supplementation continued until a scheduled first antenatal visit (ANC1), which was the trial primary end-point.

#### Non-pregnant cohort

At enrolment demographic data and medical/obstetric histories were recorded and women were clinically examined. Height (nearest mm), weight (nearest 100 g), and mid-upper arm circumference (mm; MUAC) were measured in duplicate. A venous blood sample (5 mL) was collected for later iron biomarker assessments. Two self-taken swabs were requested for a BV slide and pH measurement. Samples were not collected during menses. Women symptomatic for *T. vaginalis* and BV were treated with single dose metronidazole (2 g orally), and those symptomatic for *C. albicans* with miconazole (200 mg intravaginally, daily for 3 days). All participants received a single dose of albendazole (400 mg) and praziquantel (1500–2400 mg according to height).

Participants were individually randomised to receive either a capsule containing ferrous gluconate (60 mg) and folic acid (2.8 mg), or an identical capsule containing folic acid alone (2.8 mg), as then recommended by WHO [15]. This regimen was directly observed at weekly visits and continued for up to 18 months, when an end assessment (FIN) was completed and repeat vaginal swabs for BV and vaginal pH were requested. At FIN, duplicate swabs were additionally requested for preparation of vaginal fluid eluates for microbiota and *T. vaginalis* PCR assays. Swabs were kept cool until returned to the laboratory within 2–4 hours. A venous blood sample (5 mL) was obtained for iron biomarkers and malaria microscopy.

Women were monitored for pregnancy at weekly visits and symptoms of illness were recorded by a field worker. Symptomatic participants were directed to attend a health centre for free medical treatment for participant care, as this was required for a safety trial. Health centre staff recorded all visits, and a medically qualified researcher collated information on symptoms, clinical diagnoses and treatments. Pregnant women were instructed to attend Nanoro hospital for ANC1.

#### Pregnant cohort

At ANC1 (primary endpoint), scheduled at 13–16 weeks’ gestation, a venous blood sample (5 mL) was obtained for iron biomarkers and malaria microscopy, and self-taken vaginal swabs requested as for the non-pregnant cohort. An ultrasound examination was completed to date gestational age. Symptomatic women were treated for BV and *T. vaginalis* with metronidazole 500 mg orally twice daily for 7 days, for *C. albicans* with intravaginal miconazole 200 mg daily for 3 days, and for *N. gonorrhoeae* and *C. trachomatis* with ceftriaxone 250 mg intramuscularly once and amoxycillin 500 mg orally thrice daily for 7 days for suspected cervical infection. Women were encouraged to deliver at their closest health centre. The study provided free obstetric care.

### Laboratory procedures

#### Bacterial vaginosis

BV slides were fixed, Gram stained, air dried and forwarded to the Microbiology Laboratories, Central Manchester University Hospital NHS Trust, UK, for Nugent scoring [21]. Approximately 10% of slides, plus indeterminate slides, were read in duplicate. Nugent scores of 7–10 indicated BV, 4–6 intermediate, and 0–3 normal flora. Gram stains were available for 80.5% of women at enrolment, 76.5% at FIN, and 94.6% at ANC1, with no differences between trial arms.

#### Vaginal eluates

Each tube containing a swab for vaginal eluate was processed on laboratory arrival when 5 mL of PBS was added to the tube and shaken at high speed (5 minutes), before pipetting and freezing (–20 °C). Vaginal eluates were air freighted on dry ice to the University of Northumbria, UK.

#### Malaria microscopy

Blood films were stained with Giemsa and read independently by two qualified microscopists and, in the case of discordant results, by a third reader.

#### Iron status

At the Nanoro Research Laboratories, ferritin, indicative of iron stores, and serum transferrin receptor (sTfR), indicative of functional iron deficit, were measured in duplicate by ELISA (Spectro Ferritin S-22 and TFC 94 Transferrin Receptor, RAMCO Laboratories Inc., Texas) and C-reactive protein (CRP) by ELISA (EU59131, IBL International, GMBH, Hamburg). Definitions of iron deficiency were (1) adjusted ferritin (adjFE) allowing for inflammation, ferritin < 15 μg/L if CRP < 10 μg/mL, or ferritin < 70 μg/L if CRP ≥ 10 μg/mL; or (2) a ratio of sTfR μg/mL to log_10_ ferritin > 5.6, [22], which assesses both stored and functional iron and is possibly less affected by inflammation.

#### Microbiota/T. vaginalis qPCR

DNA was extracted from vaginal eluates using the PowerLyzer Power Soil kit (MoBio, SD, USA) with the following modifications. A 250-μL aliquot of vaginal eluate was combined with 500 μL of bead solution added to the bead tube and processed following the manufacturer’s instructions. DNA extraction negative controls were processed for each kit and sequenced alongside test samples. Bacterial profiling of the variable region 4 (V4) of the 16S rRNA gene was performed by NU-OMICS (Northumbria University) based on the Schloss wet-lab MiSeq procedure [23], and *T. vaginalis* by qPCR [24] (Additional file 2).

### Statistical analysis

The sample size was determined from formal power calculations for the malaria trial endpoints [25]. Analyses excluded women who remained non-menarcheal throughout the trial and any who became menarcheal within 6 months of FIN when menses may have been irregular. The primary analyses were comparisons of BV (Nugent > 6) risk at ANC1 and at FIN (menarcheal) by arm on an intention to treat basis using a binomial model with a log link, and baseline infection and use of antibiotics in the 3 months prior to assessment as covariates. Other infection markers were analysed similarly. Nugent scoring gave a 3-level classification and each level was compared to the remaining levels. Intention to treat analyses of iron deficiency used a similar approach with no covariates.

Associations between baseline infections and dichotomised iron deficiency markers were similarly analysed, but with no covariate for antibiotic use, as free study prescriptions had not commenced. The analysis adjusted for MUAC, a surrogate for nutritional status. Associations between baseline infection indicators and continuous biomarkers followed similar binomial models, with log-transformed values scaled by their standard deviations to give comparable risk ratios across markers. The *P* values for multiple markers were adjusted for multiple testing using the false discovery rate method [26].

Receipt of antibiotics, antifungals and analgesics was classified by clinical indication. Poisson regression models were used to estimate incidence ratios and to test for differences between arms, using number of visits between enrolment and assessment as an exposure time offset. Incidence rates are presented per 50 visits as this approximated the mean number of visits prior to follow-up assessment. For separate indications, *P* values were adjusted using the false discovery rate approach.

The statistical methods for analysis and visualisation of microbiota communities are described in Additional file 2. Multinomial regression models were used to identify if Community State Type (CST) was associated with trial arm, pregnancy, iron deficiency or infection markers.

## Results

### Participants

**Fig. 1:**
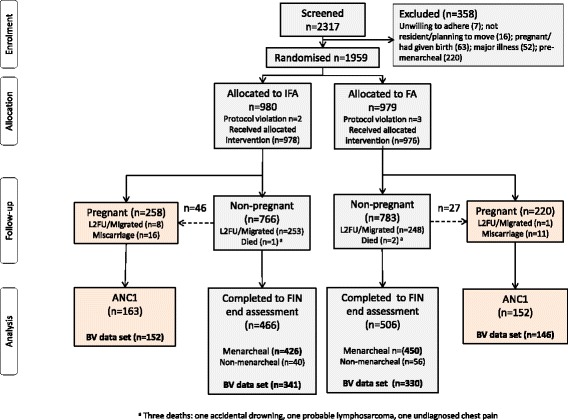
Participant flow chart

A total of 1959 nulliparous women were randomised and 1954 were included in the intention to treat dataset (Fig. 1). During follow-up, 478 (24.5%) women became pregnant, with 1476 remaining non-pregnant; 315 (65.9%) were assessed at ANC1, with two-thirds of these visits occurring at < 20 weeks’ gestation. Among the non-pregnant cohort, defined as menarcheal and followed up to 18 months, 877 (56.6%) women completed an end assessment survey (FIN). Women lost to follow-up differed in some baseline characteristics (Additional file 3: Table S1). Table 1 shows baseline participant characteristics, categorised by subsequent status in constituting the pregnant or non-pregnant cohorts. Trial arms were well balanced. In the intention to treat analysis, the median (interquartile range) number of directly observed treatments as a percentage of the number of weeks in the study was 79% (58–90%, n = 1954), with no difference by trial arm. For the total sample, adolescents (<20 years) comprised 93%. BV prevalence was 12.3%, with 8.9% having intermediate flora. BV correlated with MUAC (RR per cm 1.23; 95% CI 1.08–1.41, *P* = 0.003) and BMI (RR per kg/m^2^ 1.23; 95% CI 1.08–1.40, *P* = 0.002), but not with age (*P* = 0.82). Prevalence of vaginal discharge was 1.6%. Vaginal pH ≥ 4.5 was recorded in 50.6%, with no difference between arms.

**Table 1:** Baseline characteristics by trial arm of participants who subsequently became pregnant or remained non-pregnant during follow-up

Variable	Pregnant cohort			Non-pregnant cohort		
	N^a^	Iron	Control	N^a^	Iron	Control
		N = 258	N = 220		N = 766	N = 783
Sociodemographic						
Age, years, mean (SD)	478	17.1 (1.7)	17.1 (1.7)	1549	16.7 (1.7)	16.7 (1.8)
Ethnic group, n (%)						
Mossi	478	250 (96.9)	214 (97.3)	1548	730 (95.3)	757 (96.8)
Other	478	8 (3.1)	6 (2.7)	1548	36 (4.7)	25 (3.2)
Marital status						
Married	478	22 (8.5)	15 (6.8)	1544	17 (2.2)	18 (2.3)
Never married	478	236 (91.5)	204 (92.7)	1544	748 (97.8)	760 (97.6)
Previously married	478	0 (0)	1 (0.5)	1544	0 (0)	1 (0.1)
Occupation,^b^ n (%)						
Student	478	60 (23.3)	51 (23.2)	1549	255 (33.3)	274 (35.0)
Trading	478	10 (3.9)	10 (4.5)	1549	24 (3.1)	21 (2.7)
Domestic labour	478	154 (59.7)	120 (54.5)	1549	401 (52.3)	407 (52.0)
Farming	478	130 (50.4)	101 (45.9)	1549	269 (35.1)	290 (37.0)
Other	478	3 (1.2)	3 (1.4)	1549	3 (0.4)	2 (0.3)
Education, n (%)						
No schooling	477	176 (68.5)	142 (64.5)	1544	447 (58.6)	450 (57.6)
Primary	478	54 (20.9)	38 (17.3)	1549	168 (21.9)	169 (21.6)
Lower secondary	477	24 (9.3)	38 (17.3)	1544	144 (18.9)	153 (19.6)
Higher secondary	477	3 (1.2)	2 (0.9)	1544	4 (0.5)	9 (1.2)
Literate, n (%)	473	68 (26.7)	62 (28.4)	1530	278 (36.8)	297 (38.4)
Reproductive history and infections, n (%)						
Menarcheal	478	241 (93.4)	203 (92.3)	1549	639 (83.4)	645 (82.4)
Menarche age, mean (SD)	443	14.9 (1.0)	14.8 (1.1)	1279	15 (1.0)	15.1 (1.1)
Ever had sex	478	98 (38)	82 (37.3)	1548	159 (20.8)	164 (21.0)
Uses contraception	478	54 (20.9)	51 (23.2)	1549	106 (13.8)	86 (11.0)
Uses condoms	478	53 (20.5)	50 (22.7)	1549	103 (13.4)	81 (10.3)
Nugent 7–10	391	33 (15.7)	23 (12.7)	1247	80 (13.0)	68 (10.8)
Nugent 4–6	391	17 (8.1)	11 (6.1)	1247	49 (8.0)	66 (10.5)
Nugent 0–3	391	160 (76.2)	147 (81.2)	1247	487 (79.1)	497 (78.8)
Vaginal discharge	478	3 (1.2)	4 (1.8)	1549	13 (1.7)	12 (1.5)
Vaginal pH ≥ 4.5	394	119 (55.9)	84 (46.4)	1280	317 (50.3)	328 (50.5)
Nutrition and iron biomarkers, n (%)						
Drinks alcohol	476	140 (54.5)	119 (54.3)	1549	372 (48.6)	380 (48.5)
BMI, kg/m^2^, mean (SD)	478	20.2 (1.8)	20.3 (2.1)	1549	19.7 (2.2)	19.6 (2.2)
MUAC, cm, mean (SD)	478	24.1 (1.8)	24.3 (2.2)	1549	23.6 (2.1)	23.6 (2.2)
Iron deficient (adjFE)	467	25 (10.0)	26 (12.0)	1528	84 (11.1)	100 (13.0)
Iron deficient (ratio sTfR/log ferritin)	470	52 (20.6)	55 (25.3)	1524	162 (21.4)	168 (21.9)
Adherence to treatment						
DOT,^c^ mean (SD)	478	70.3 (27.0)	71.4 (26.0)	1549	70.0 (27.8)	70.8 (26.3)

^a^Total responders

^b^More than one response allowed

^c^Number of directly observed treatments as a percentage of the number of weeks from enrolment to assessment

*adjFE* adjusted ferritin, *BMI* body mass index, *DOT* directly observed treatment, *MUAC* mid-upper arm circumference, *SD* standard deviation, *sTfR* serum transferrin receptor

### Infections and iron status at baseline assessment

Baseline prevalence of iron deficiency was lower using the single iron biomarker adjFE than when using the combined marker sTfR/log ferritin, but with either marker, it did not differ by trial arm (Table 1). Prevalence of iron deficiency remained very similar by trial arm using varying CRP cut-offs (5–25 μg/mL) (Additional file 4b). Iron replete participants at baseline were less likely to have normal vaginal flora (Nugent score 0–3) (adjFE: *P* = 0.036; sTfR/log ferritin ratio: *P* = 0.255). Vaginal discharge was reported by only a few women but was less prevalent with iron repletion (adjFE: *P* = 0.03; sTfR/log ferritin ratio: *P* = 0.06) (Table 2). A sensitivity analysis using alternative lower ferritin thresholds of < 15 μg/L and CRP < 10 μg/mL, or < 30 μg/L and CRP ≥ 10 μg/mL, for iron depletion adjusting for inflammation was explored to assess whether a lower ferritin cut-off significantly altered these associations (Additional file 3: Table S2). Prevalence of vaginal discharge remained significantly lower in iron replete women (*P* = 0.013), and normal vaginal flora remained significantly less frequent in iron replete women (*P* = 0.028). High pH was positively associated with plasma ferritin (adjFE) (*P* = 0.015 adjusted for MUAC), but not the sTfR/log ferritin ratio (*P* = 0.14) (Additional file 3: Table S3).

**Table 2:** Baseline host iron status using two definitions of iron deficiency and infection markers (n = 1673, excluding 281 non-menarcheal)

Infection	N	Replete (%)	Deficient (%)	Unadjusted		Adjusted^a^		
				RR (95% CI)	*P* value^b^	N^c^	RR (95% CI)	*P* value
Adjusted ferritin								
BV^d^	1347				0.127	1326		0.032
Nugent 7–10^e^	1347	142/1151 (12.3)	15/175 (8.6)	1.44 (0.87–2.39)	0.168	1326	1.53 (0.92–2.54)	0.104
Nugent 4–6^e^	1347	108/1151 (9.4)	11/175 (6.3)	1.49 (0.82–2.72)	0.203	1326	1.47 (0.81–2.68)	0.210
Nugent 0–3^e^	1347	901/1151 (78.3)	149/175 (85.1)	0.92 (0.86–0.99)	0.036	1326	0.92 (0.86–0.98)	0.016
Vaginal discharge	1673	21/1430 (1.5)	8/214 (3.7)	0.39 (0.18–0.88)	0.044	1644	0.41 (0.18–0.92)	0.030
pH ≥ 4.5	1371	608/1166 (52.1)	86/183 (47.0)	1.11 (0.94–1.31)	0.204	1349	1.11 (0.94–1.30)	0.222
sTfR/log ferritin ratio > 5.6								
BV^d^	1347				0.513	1324		0.224
Nugent 7–10^e^	1347	127/1030 (12.3)	30/294 (10.2)	1.21 (0.83–1.76)	0.358	1324	1.25 (0.86–1.83)	0.239
Nugent 4–6^e^	1347	95/1030 (9.2)	24/294 (8.2)	1.13 (0.74–1.73)	0.644	1324	1.12 (0.73–1.71)	0.619
Nugent 0–3^e^	1347	808/1030 (78.4)	240/294 (81.6)	0.96 (0.90–1.02)	0.255	1324	0.96 (0.90–1.02)	0.231
Vaginal discharge	1673	18/1270 (1.4)	11/372 (3.0)	0.48 (0.23–1.01)	0.070	1642	0.49 (0.23–1.04)	0.062
pH ≥ 4.5	1371	551/1044 (52.8)	144/303 (47.5)	1.11 (0.97–1.27)	0.117	1347	1.11 (0.97–1.26)	0.126

^a^Adjusted for MUAC

^b^Fishers Exact Test

^c^Number of observations in adjusted analysis

^d^Global test (Fishers/ordinal regression) for BV as 3-category outcome

^e^Each group compared to both other groups

*BV* bacterial vaginosis, *sTfR* serum transferrin receptor

### Lower genital tract infection in the pregnant cohort

#### ANC1

Prevalence of iron deficiency (adjFE) did not differ significantly between trial arms (6.9% (iron) and 12.7% (control); RR 0.54, 95% CI 0.27–1.1, *P* = 0.12) or based on the sTfR/log ferritin ratio (11.1% (iron) and 12.7% (control); RR 0.88, 95% CI 0.48–1.61, *P* = 0.73).

Prevalence was similar in iron and control arms (Table 3) for BV (7.9% vs. 6.2%; RR 0.93, 95% CI 0.33–2.56, *P* = 0.88), intermediate flora (11.2% vs. 13.0%; *P* = 0.86), *T. vaginalis* (15.6% vs. 10.1%; *P* = 0.17), and vaginal discharge (8.6% vs. 7.9%; *P* = 0.85). Abnormal discharge occurred in 24% of women with BV and 8% of those with *T. vaginalis*.

**Table 3:** Lower genital tract infection indicators in pregnant (ANC1) and non-pregnant (FIN) cohorts by trial arm

Assessment	Infection marker	N	Trial arm		Unadjusted		Adjusted^a^		
			Iron (%)	Control (%)	RR (95% CI)	*P* value^b^	N^c^	RR (95% CI)	*P* value^c^
Pregnant	Bacterial vaginosis^d^	298				0.776	245		0.704
ANC1	Nugent 7–10^e^	298	12/152 (7.9)	9/146 (6.2)	1.28 (0.56–2.95)	0.653	245	0.93 (0.33–2.56)	0.881
N = 315	Nugent 4–6^e^	298	17/152 (11.2)	19/146 (13.0)	0.86 (0.47–1.59)	0.723	245	0.94 (0.47–1.88)	0.858
	Nugent 0–3^e^	298	123/152 (80.9)	118/146 (80.8)	1.00 (0.90–1.12)	1	245	1.03 (0.91–1.15)	0.665
	*T. vaginalis* ^f^	286	23/147 (15.6)	14/139 (10.1)	1.55 (0.83–2.90)	0.217	286	1.55 (0.83–2.90)	0.168
	Vaginal discharge	315	14/163 (8.6)	12/152 (7.9)	1.09 (0.52–2.28)	0.841	315	1.08 (0.51–2.25)	0.846
	pH ≥ 4.5	309	92/158 (58.2)	86/151 (57.0)	1.02 (0.84–1.24)	0.908	260	1.06 (0.86–1.31)	0.555
Non-pregnant	Bacterial vaginosis^d^	671				0.588	546		0.676
FIN^f^	Nugent 7–10^e^	671	46/341 (13.5)	40/330 (12.1)	1.11 (0.75–1.65)	0.645	546	0.86 (0.56–1.34)	0.509
N = 877	Nugent 4–6^e^	671	35/341 (10.3)	26/330 (7.9)	1.30 (0.80–2.11)	0.347	546	1.21 (0.72–2.03)	0.467
	Nugent 0–3^e^	671	260/341 (76.2)	264/330 (80.0)	0.95 (0.88–1.03)	0.263	546	0.99 (0.90–1.08)	0.806
	*T. vaginalis* ^f^	716	20/360 (5.6)	15/356 (4.2)	1.32 (0.69–2.53)	0.489	716	1.30 (0.67–2.50)	0.432
	Vaginal discharge	874	49/425 (11.5)	31/449 (6.9)	1.67 (1.09–2.57)	0.019	874	1.63 (1.06–2.51)	0.026

^a^Adjusted for baseline infection and use of antibiotics in previous 3 months

^b^Fishers exact test

^c^Logistic regression

^d^Each group compared to both other groups

^e^Excludes those less than 6 months post menarcheal

^f^Baseline not available

### Lower genital tract infection in the non-pregnant cohort

#### FIN

Prevalence of iron deficiency (adjFE) was 9.0% (iron) and 11.0% (control) (RR 0.82, 95% CI 0.55–1.22, *P* = 0.37) and, based on sTfR/log ferritin ratio, it was 20.1% (iron) and 21.2% (control) (RR 0.95, 95% CI 0.73–1.23, *P* = 0.74).

No prevalence difference was observed for BV (13.5% iron vs. 12.1% control, RR 0.86, 95% CI 0.56–1.34, *P* = 0.51), intermediate flora (10.3% vs. 7.9%, *P* = 0.47), or *T. vaginalis* (5.6% vs. 4.2%, *P* = 0.43) (Table 3). Vaginal discharge was more frequent in iron-supplemented women (11.5% vs. 6.9%, *P* = 0.026). This difference would be considered non-significant allowing for the number of secondary outcomes tested. Abnormal discharge occurred in 9% of those with BV and 18% with *T. vaginalis*.

### Comparison of non-pregnant and pregnant cohorts

Cross-sectional BV prevalence was higher in non-pregnant women at FIN (12.8%) than at ANC1 (7.0%) (RR 1.8, 95% CI 1.2–2.9, *P* = 0.011, adjusted for antibiotic use), but *T. vaginalis* prevalence was lower (4.9%) (RR 0.38, 95% CI 0.24–0.59, *P* < 0.001). *P. falciparum* prevalence was 54% at ANC1 and 41% at FIN, despite free access to anti-malarial treatments.

### Antibiotic courses

The number of antibiotic courses (excluding anti-malarials) provided to the non-pregnant cohort was higher in women receiving iron supplements (*P* = 0.014) (Table 4). These also received more anti-fungal treatments (*P* = 0.014) and analgesics (*P* = 0.008). BV and *T. vaginalis* were mostly asymptomatic and, as these infections were unlikely to account for the high proportion of women receiving antibiotics, all indications for antibiotic prescription at Health Centres were assessed (Table 5). The most frequent indications were respiratory illness, gastrointestinal, reproductive tract and local infections. Gastrointestinal infections were treated more frequently in iron-supplemented women (*P* = 0.005, allowing for the multiple indications tested) (Table 5). With far fewer events, no significant differences were detected in the pregnant cohort at ANC1 (Additional file 3: Table S4).

**Table 4:** Health Centre treatment courses prescribed to pregnant and non-pregnant cohorts by trial arm from enrolment to ANC1 (pregnant) or FIN (non-pregnant)

Treatment	Number of treatments		Mean treatments per person		Mean treatments per 50 visits^a^		Incidence ratio^b^ (95% CI)	*P* value
	Iron	Control	Iron	Control	Iron	Control		
Pregnant			n = 163	n = 152	Visits = 6907	Visits = 6235		
Antibiotics	92	73	0.564	0.480	0.666	0.585	1.14 (0.84–1.55)	0.411
Anti-fungals^c^	16	15	0.098	0.099	0.116	0.120	0.96 (0.47–1.95)	0.916
Analgesics	107	107	0.656	0.704	0.775	0.858	0.90 (0.69–1.18)	0.454
Non-pregnant			n = 426	n = 451	Visits =26473	Visits =27633		
Antibiotics	331	284	0.777	0.630	0.625	0.514	1.22 (1.04–1.43)	0.015
Anti-fungals^c^	39	21	0.092	0.047	0.074	0.038	1.94 (1.14–3.30)	0.014
Analgesics	481	421	1.129	0.933	0.908	0.762	1.19 (1.05–1.36)	0.008

^a^Mean number of visits: 42 in pregnant and 63 in non-pregnant

^b^Incidence ratio was computed using Poisson regression with the number of visits as the exposure period

^c^More than 90% of vaginal pessaries prescribed for genital tract infections

**Table 5:** Mean number of health centre antibiotic treatments in non-pregnant cohort by infection diagnosis and trial arm from enrolment to FIN

Infection indication	Iron	Control	Iron per 50 visits n = 26,473	Control per 50 visits n = 27,633	Incidence ratio^a^ (95% CI)	*P* value	PFDR^b^
Malaria^c^	41	28	0.077	0.051	1.53 (0.94–2.47)	0.084	0.278
Respiratory	75	87	0.142	0.157	0.90 (0.66–1.23)	0.503	0.719
Local^d^	75	57	0.142	0.103	1.37 (0.97–1.94)	0.071	0.278
Gastrointestinal^e^	62	30	0.117	0.054	2.16 (1.39–3.34)	<0.001	0.005
Urinary tract^f^	10	9	0.019	0.016	1.16 (0.47–2.86)	0.747	0.934
STI^g^	13	7	0.025	0.013	1.94 (0.77–4.86)	0.158	0.292
Genital^h^	15	16	0.028	0.029	0.98 (0.48–1.98)	0.952	0.993
Dental	11	5	0.021	0.009	2.30 (0.80–6.62)	0.123	0.292
Meningitis	0	3	0	0.005	0 (NA)	0.993	0.993
Miscellaneous^i^	29	42	0.055	0.076	0.72 (0.45–1.16)	0.175	0.292

^a^Incidence ratio was computed using Poisson regression with the number of visits as the exposure period

^b^ *P* value adjusted for multiple testing using false discovery rate method [26]

^c^Antibiotics in addition to anti-malarial treatment

^d^Non-enteric and non-respiratory, includes ear, skin, ophthalmic, abscess, wounds, local trauma, sinusitis, mastoiditis, keloid, scalp ringworm

^e^Dysentry, typhoid, enteritis, intestinal parasitosis, gastric ulcer, gastroenteritis, diarrhoea and abdominal pain, vomiting and abdominal pain, amoebiasis, colopathy, sub-occlusion

^f^Cystitis, dysuria

^g^Includes syphilis, trichomoniasis

^h^Upper or lower genital infection

^i^Itching, colic, headache, generalised or localised pain, fever, vomiting only, anaemia, anxiety, neuralgia, spasms, urticarial, plus non-infectious specific diagnoses: cancers, fertility problems, angina, self-medication, allergy, burns, renal stones, contraception, thyroid and cardiac diseases, epilepsy, filariasis, foreign body, venomous bites, fractures, migraine, HIV, varicella, mumps, prophylactic and non-classifiable: includes three uncertain descriptors, or absent statement

*PFDR* positive false discovery rate, *STI* sexually transmitted infection

### Vaginal microbiota

**Fig. 2:**
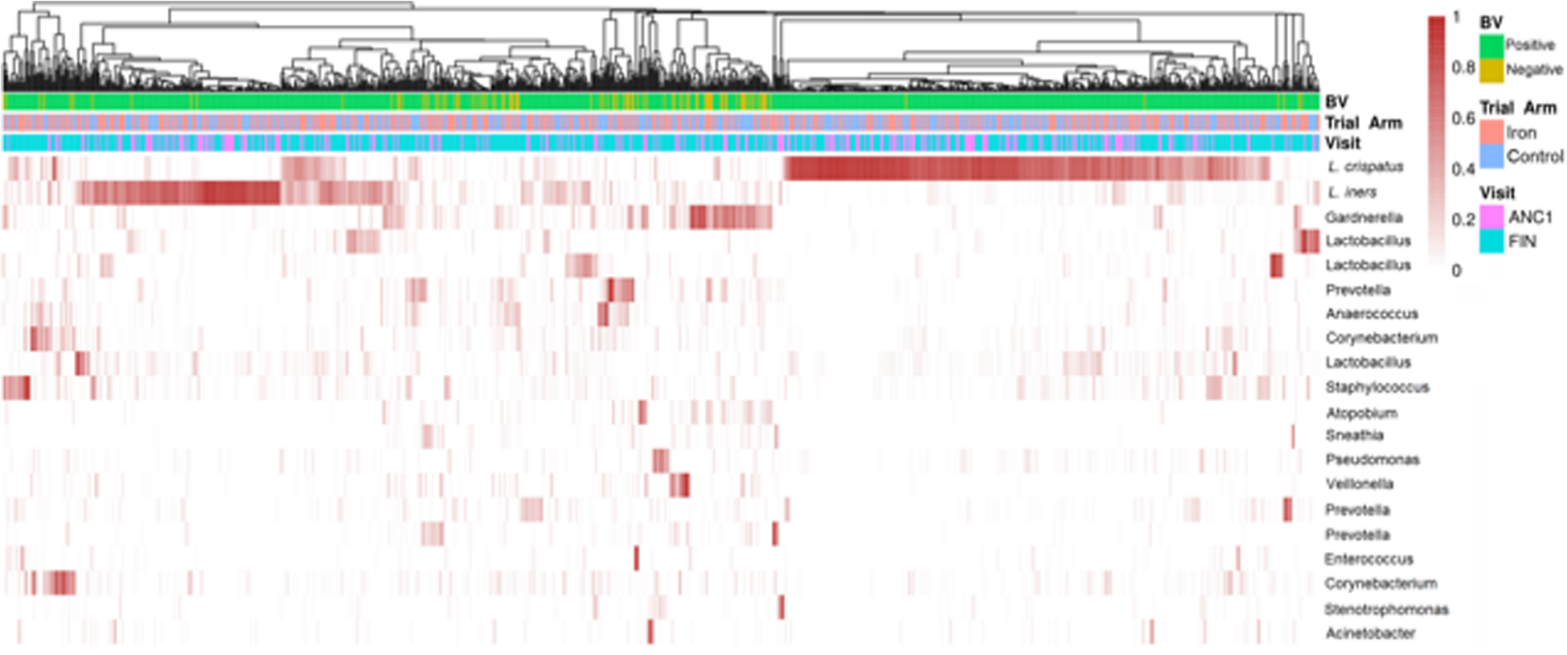
Heatmap showing distribution of the 20 most abundant taxa across all samples with coloured bars showing trial arm, study visit and BV infection

Figure 2 demonstrates the distribution of the top 20 taxa for all samples combined by trial arm, study visit and BV infection. Three CSTs typified this population, corresponding to CST I (40.4%), III (24.6%) and IV (35%), dominated by *Lactobacillus crispatus*, *Lactobacillus iners* and a mixed community with reduced lactobacilli, respectively. CST IV was more frequent in the non-pregnant cohort at FIN than pregnant women at ANC1 (*P* < 0.001). CST IV was associated with BV (*P* < 0.001) and *T. vaginalis* (*P* < 0.001) (Table 6). No differences were observed in CST IV frequency or alpha- and beta-diversity, by trial arm, or host iron status, in pregnant or non-pregnant cohorts (Additional file 4c). Iron replete women at ANC1 and FIN had non-significantly higher Shannon diversity. CST frequencies did not differ significantly from earlier results using the lower ferritin threshold (Additional file 3: Table S5).

**Table 6:** Community state type distribution in pregnant and non-pregnant (menarcheal) cohorts by trial arm, iron deficiency and infection status

Variable	Visit	Group	n	CST I n (%)	CST III n (%)	CST IV n (%)	*P* value^a^
Trial arm	ANC1	Iron	144	70 (48.6)	39 (27.1)	35 (24.3)	0.518
		Control	136	61 (44.9)	45 (33.1)	30 (22.1)	
	FIN^b^	Iron	349	121 (34.7)	88 (25.2)	140 (40.1)	0.113
		Control	341	140 (1.1)	66 (19.4)	135 (9.6)	
Iron deficiency, Adjusted ferritin	ANC1	No	249	118 (47.4)	75 (30.1)	56 (22.5)	0.57
		Yes	27	10 (37.0)	9 (33.3)	8 (29.6)	
	FIN^b^	No	620	237 (38.2)	140 (22.6)	243 (39.2)	0.281
		Yes	65	20 (30.8)	13 (20.0)	32 (49.2)	
Ratio sTfR/log ferritin	ANC1	No	247	120 (48.6)	74 (30.0)	53 (21.5)	0.216
		Yes	32	11 (34.4)	10 (31.2)	11 (34.4)	
	FIN^b^	No	544	205 (37.7)	124 (22.8)	215 (39.5)	0.81
		Yes	140	52 (37.1)	29 (20.7)	59 (42.1)	
Nugent score^c^	ANC1	7-10	15	0 (0)	1 (6.7)	14 (93.3)	<0.001
		4-6	31	7 (22.6)	6 (19.4)	18 (58.1)	<0.001
		0-3	221	116 (52.5)	74 (33.5)	31 (14.0)	<0.001
	FIN^b^	7-10	80	2 (2.5)	6 (7.5)	72 (90.0)	<0.001
		4-6	60	11 (18.3)	6 (10.0)	43 (71.7)	<0.001
		0-3	501	232 (46.3)	132 (26.3)	137 (27.3)	<0.001
*T. vaginalis*	ANC1	No	243	121 (49.8)	73 (30.0)	49 (20.2)	0.006
		Yes	37	10 (27.0)	11 (29.7)	16 (43.2)	
	FIN^b^	No	655	260 (39.7)	149 (22.7)	246 (37.6)	<0.001
		Yes	35	1 (2.9)	5 (14.3)	29 (82.9)	
High pH ≥ 4.5	ANC1	No	123	73 (59.3)	37 (30.1)	13 (10.6)	<0.001
		Yes	153	58 (37.9)	45 (29.4)	50 (32.7)	
Vaginal discharge	ANC1	No	260	122 (46.9)	80 (30.8)	58 (22.3)	0.268
		Yes	20	9 (4.5)	4 (20.0)	7 (35.0)	
	FIN^b^	No	618	232 (37.5)	144 (23.3)	242 (39.2)	0.205
		Yes	69	28 (40.6)	10 (14.5)	31 (44.9)	
Antibiotics within previous three months	ANC1	No	252	118 (46.8)	73 (29.0)	61 (24.2)	NA
		Yes	28	13 (46.4)	11 (39.3)	4 (14.3)	
	FIN^b^	No	616	236 (38.3)	136 (22.1)	244 (39.6)	NA
		Yes	74	25 (33.8)	18 (24.3)	31 (41.9)	

^a^ *P* value for association between status and CST from a multinomial regression model adjusting for antibiotic use in the 3 months prior to assessment

^b^Menarcheal women only

^c^Each Nugent group compared to the other two. BV corresponds to Nugent score 7–10

*ANC1* first antenatal visit, *CST* community state type, *FIN* end assessment, *sTfR* serum transferrin receptor

## Discussion

In this young population, reported sexual activity was low at recruitment but its commencement carried a substantial risk of immediate pregnancy, a sexually transmitted infection (*T. vaginalis*), an unstable microbiota and malaria, which was more frequent in primigravidae. Evidence on effects of intermittent iron supplementation on infectious disease outcomes is scarce and unclear, with few studies and small sample sizes [15]. This randomised, controlled, double blind trial found no evidence that 60 mg iron/2.8 mg folic acid supplements offered weekly, increased the risk of BV, *T. vaginalis*, or CST IV microbiota in either the pregnant or non-pregnant cohort. A two-fold increased use of antibiotics for treatment of gastrointestinal infections, with increased use of anti-fungals for lower genital infection in the non-pregnant iron supplemented cohort, raises new concerns about the safety of weekly iron supplements for young women. Nutritional status, reflected by MUAC, was an effect modifier for BV infection. This result adds to a growing body of evidence of an association between diet and BV, for which there is currently no clear explanation [27].

A major strength of this study was its large cohort of non-pregnant peri-menarcheal adolescents, as well as a concurrent pregnancy cohort [16]. Some loss to follow-up occurred due to movement outside the study area following marriage, unwanted pregnancy or, if unmarried, working for relatives living elsewhere. Trial participants were young, nulliparous girls, with low prevalence of iron deficiency at recruitment. Accurate estimation of iron deficiency prevalence is influenced by effects of inflammation on indices such as ferritin. A recent meta-analysis in women of reproductive age experiencing inflammation estimated the difference of depleted iron stores between adjusted and unadjusted ferritin values as 2–8 median percentage points, dependent on the adjustment method [28]. We used a high ferritin cut-off of < 70 μg/L for iron deficiency in women with CRP ≥ 10 μg/mL, allowing for inflammation, as well as the lower cut-off < 15 μg/L with CRP < 10 μg/L to include those without inflammation. CRP concentration increases in early pregnancy, which was accommodated by using the CRP < 10 μg/L cut-off. Although prevalence of iron deficiency in absolute terms varied according to the CRP cut-off used, sensitivity analyses showed that trends and trial arm differences in iron deficiency were similar irrespective of CRP level. We additionally used the sTfR/log_10_ ferritin ratio > 5.6, which is derived from an intermediate ferritin cut-off of < 30 μg/L [22]. We considered this provided a reasonable estimate of iron deficiency. Sensitivity analysis of alternative ferritin thresholds for defining iron deficiency, adjusting for inflammation, gave very similar results.

Despite good supplementation adherence over an 18 month period, facilitated by weekly direct observation, there was no significant improvement in systemic iron biomarkers. The absence of an association between iron supplementation and BV or *T. vaginalis* prevalence could relate to the lack of intervention efficacy in reducing iron deficiency. At baseline, iron-replete participants were less likely to have normal vaginal flora, which suggests host iron status is reflected in the mucosa. Following supplementation, an increased prescription for fungal infections was also observed inferring an iron-fungal infection interaction [29]. Antibiotics used for treatment of enteric infections may have altered lower genital tract bacterial profiles. Cessation of menses with pregnancy would also reduce iron availability to haem-utilising pathogens.

Chronic inflammation from malaria, which was frequent, as well as other infections, would increase hepcidin release, restricting iron absorption. In Beninese women with afebrile *P. falciparum* parasitaemia, dietary iron absorption was reduced by approximately 40% with infection [30]. In the non-pregnant cohort, a significantly higher number of antibiotic-treated gastrointestinal infections – predominantly diagnosed as dysentery, typhoid, and intestinal parasitosis – occurred in supplemented women, possibly related to unabsorbed iron becoming available to colonic gut microbiota [30]. Although some misclassification of infection categories would arise due to a lack of laboratory confirmation, this should not alter findings by trial arm. Increased gut inflammation and pathogenicity following iron supplementation, or fortification, is reported in children exposed to high infection pressure in Ghana [31] and Kenya [32], and increased risk of bloody dysentery and respiratory infection in Pakistan [33]. This is the first report in young women to show increased enteric symptoms requiring antibiotic treatments following iron supplementation. Concurrent infection with non-typhi salmonella and *P. falciparum* should be considered [34]. Increased use of antifungal prescriptions in the iron-supplemented non-pregnant cohort is of interest because, both in the gut and vaginal mucosa, *C. albicans* is a normal commensal, competing with other organisms for nutrients, and increased fungal virulence due to iron malabsorption could be anticipated. In addition, disruption to normal bacterial gut populations due to tetracycline treatment can result in candida overgrowth, reducing colonisation resistance [35]. The intestinal tract is considered as a reservoir of infection leading to recurrent vaginal candidiasis based on the correspondence between vaginal and stool samples [36].

Self-taken vaginal swabs proved acceptable, providing samples for gram stains, vaginal eluates and microbiota assays. The absence of differences in microbiota CST categories by trial arm is consistent with no supplementation effect on BV or *T. vaginalis*. BV was associated with CST IV, and both had lower prevalence in pregnant than non-pregnant women [37]. This pregnancy difference is probably related to hormonal factors [38], although we cannot exclude the possibility that it is mediated by differences in other characteristics between those who became pregnant and those that did not. In marked contrast, *T. vaginalis* prevalence, also associated with CST IV, was three-fold higher at ANC1 compared to non-pregnant women at FIN, probably due to more regular sexual activity with male partners untreated for this sexually transmitted infection. Interactions between BV and *T. vaginalis* are poorly understood [39] but, under experimental conditions, *T. vaginalis* is associated with reduced populations of *Lactobacillus spp*, but not BV spp [40]. Co-infection in early pregnancy may hinder transition to stable microbiome communities, such as CST I, which is associated with better pregnancy outcomes [41]. Clinical outcomes may depend on initial host iron status, as experimental studies have shown altered inflammatory responses, higher abundance of *Bifidobacteriaceae* and *Lactobacillaceae*, and prolonged intestinal nematode survival in animals receiving iron-deficient diets [42].

At baseline, elevated vaginal pH values (≥4.5) were observed in iron-replete participants and were positively associated with plasma ferritin (*P* = 0.015). High vaginal pH may be attributable to normal pubertal changes [43], young age, irregular menses [44], and factors impacting on lactobacilli or other lactic acid producing microbes [45], but also to genital tract infection. The reason in this study for the significant association of high pH and more disturbed flora in adolescents with better iron stores before supplementation is unknown and warrants further investigation.

## Conclusions

There was no evidence that weekly iron increased the risk of BV or *T. vaginalis* infections, but the intervention was considered poorly absorbed as systemic iron biomarkers were not significantly changed. Iron supplementation did result in more gastrointestinal morbidity, leading to increased antibiotic prescription and genital tract infection requiring antifungal treatments in the non-pregnant cohort. This is a concern since recently revised WHO recommendations are shifting in favour of providing intermittent daily iron [46]. A daily regime could be more likely to exacerbate gut microbes and/or increase antibiotic/antifungal prescriptions. Since malaria is the most likely cause of iron malabsorption in the gut, iron supplementation studies in malaria endemic areas need to profile enteric and vaginal infections, as well as antibiotic use.

## Additional files

Additional file 1: Background data to the main RCT. (DOCX 18 kb)

Additional file 2: Detailed methods for microbiota and *T. vaginalis* qPCR profiling. (DOCX 13 kb)

Additional file 3: Tables not shown in Results. (DOCX 49 kb)

Additional file 4: (a) Legend to Additional figures; (b) CRP sensitivity analysis and iron deficiency; (c) Figure Shannon Diversity (microbiota results). (ZIP 116 kb)
