# Supplementary material for: Effects of long-term weekly iron and folic acid supplementation on lower genital tract infection – a double blind, randomised controlled trial in Burkina Faso
Source: BMC Med. 2017 Nov 23;15:206. doi: 10.1186/s12916-017-0967-5 (PMC5700548; doi:10.1186/s12916-017-0967-5)
Supplement: Supplementary file 1 — Background data to the main RCT. (DOCX 18 kb) [file 12916_2017_967_MOESM1_ESM.docx]

ADDITIONAL FILE 1: Main study procedures

*a. Study area and participants:* The Health and Demographic Surveillance System (HDSS) area was established in 2009. By December 2011, the HDSS recorded approximately 61 000 inhabitants, predominantly of Mossi origin. Health care was provided by twelve peripheral health centres and one referral hospital.

Individual participant information about the trial was provided by a study investigator who read this to women who were illiterate before signed consent (part 1) was obtained. Unmarried minor girls were accompanied by a legal representative who agreed to their participation. A second signed consent procedure (part 2) was instigated for participants who became pregnant during the trial, who agreed to attend for a study antenatal visit and to continue weekly follow-up visits.

*b. Procedures:* At trial enrolment (ENR) demographic data and a general history of past and present illnesses and obstetrical history including last menstrual period, age at menarche, sexual activity, use of contraceptive methods and current complaints were recorded. A study clinician completed a physical and clinical examination, including duplicate measurements of height (nearest mm, Minimeter, Raven Equipment Ltd, Essex, UK), weight (nearest 100gms, SECA scale), and mid-upper arm circumference (mm, MUAC). Women were not recruited if they had any significant illness at the time of screening that required hospitalization, including clinical signs of severe anaemia (conjunctival or mucosal pallor, tachycardia, respiratory distress), or a history or presence of major clinical disease likely to influence pregnancy outcome (sickle cell disease, diabetes mellitus, severe renal or heart disease, open tuberculosis, epilepsy, known HIV/AIDS infection). One self-taken vaginal sample for a BV glass slide was obtained using a cotton-tipped swab in a sterile tube (TSC, Lancs), and a second swab for pH measurement (pH indicator sticks ranged from 3.6-6.1). Women with signs and symptoms of vaginal infections were treated: for *T. vaginalis* and BV if pH > 4.5 was present with reported coloured discharge (single dose metronidazole 2 g orally) and *C. albicans* if pH was ≤ 4.5 (ie within normal range) but a white curdy discharge was reported with itching or burning symptoms (with miconazole 200 mg intravaginally, daily for 3 days). All participants received a single dose of albendazole (400mg) and praziquantel (1500-2400mg, according to the WHO dose pole) plus an insecticide treated bed net.

Following recruitment participants were individually randomized to one of two trial arms and received weekly one of identical red coloured vegetable cellulose (hypromellose) capsules containing either ferrous gluconate (60mg) and folic acid (2.8mg), or folic acid alone (2.8mg)(G and G Food Supplies Ltd, West Sussex, UK), which were provided in tamper evident opaque containers of 20 capsules. A block allocation sequence was used with randomly determined block lengths. The dose of supplements was based on recommendations made by WHO. The field workers kept one allocated container per participant for the weekly supply, with subsequent replacements from a central depository. The supplement code was unknown to investigators and was revealed after data base lock and completion of data analysis.

*c. Safety monitoring:*  After the baseline survey symptoms of illness were recorded by female field assistants (FFAs) during weekly home visits, and ingestion of supplements was directly observed. Participants not present during the weekly home visit and who could not be located for two consecutive re-visits by the FFA were reported as temporarily absent until the next weekly follow-up. Safety and tolerability was evaluated by recording and grading adverse events (AE) through the active weekly and passive follow-up. There were two levels of recording: complaints recorded by the FFA at the weekly visits (no clinical qualification needed), and AE recorded and graded through passive follow-up at the health centres or hospital (clinical qualification required). Data on serious adverse events (SAEs) were collected by active (weekly) and passive surveillance. If a women died outside hospital verbal autopsies were done by the HDSS team according to their standardized protocol. SAEs (including deaths) were reported according to the available information from FFAs and health centre staff.

*d. Non-pregnant cohort study assessments:* The dosing regimen was continued for up to18 months for the non-pregnant cohort, at which time women were referred to the study clinic for an end assessment. This included a medical history and clinical examination including duplicate anthropometric measurements of height, weight, MUAC, and blood pressure, axillary temperature, assessment of conjunctival or mucosal pallor, exploratory cardio-pulmonary examination, and assessment of hepato-splenomegaly. A venous blood sample (5ml) was collected for a malaria smear, assessment of iron and infection markers, and for storage on filter paper. In cases of fever (T°≥37.5°C) and/or history of fever in the previous 48 hours a Rapid Diagnostic Test was performed from the same venous blood sample and the enrolment treatment protocol followed. Women provided self- taken vaginal swabs and slides were made for later assessment of BV by Gram stain.

*e. Pregnant cohort study assessments:* Women who became pregnant within the 18 month follow up period and consented to enter the pregnancy cohort were referred to Nanoro hospital for a scheduled antenatal visit (standardized at about 13-16 weeks gestation according to the last menstrual period). This was termed ANC1 and was performed by one of the study nurses/doctors. The weekly supplement was withdrawn when antenatal care commenced and haematinics provided according to national policy as daily iron (60mg) and folic acid (400µg) tablets, but weekly follow-up continued. Routine antenatal care included antenatal booklet, screening for pre-eclampsia, clinical anaemia and syphilis, counselling and voluntary HIV testing and tetanus toxoid immunization. All women regardless of the malaria laboratory result received the routine first dose of the anti-malarial sulfadoxine-pyrimetrhamine (IPTp) if gestational age was >13 weeks. Women in the first trimester (≤13 weeks gestation), if malaria positive, were treated with oral quinine. Severely anaemic pregnant (Hb <7g/dL) women were referred to Nanoro hospital. At ANC1 women were treated symptomatically for BV and *T. vaginalis,* with metronidazole 500 mg orally twice daily for seven days if whiff test positive, pH ≥ 4.5 and abnormal discharge were present; for *T. vaginalis,* with metronidazole 500 mg orally twice daily for seven days, if motile trichomonads were observed in a wet mount; and for *C. albicans* with miconazole 200 mg intravaginally, daily for three days, if yeast spores or hyphae were observed in a wet mount; for *N. gonorrhoeae* and *C. trachomatis*, with ceftriaxone 250 mg intramuscularly once and amoxycillin 500 mg orally x three tablets per day for seven days if cervical infection was suspected. Women then followed routine antenatal visits in their respective health centres where they received a second dose of IPTp which was recorded in the ANC booklet. Effects of IPTp on reproductive tract infections (Chico et al, 2017) were not considered in the current analysis, the endpoint for which was ANC1 when IPTp was first administered.

Chico RM Chaponda EB, Ariti C, Chandramohan D. Sulfadoxine-pyrimethamine exhibits dose-response protection against adverse birth outcomes related to sexually transmitted and reproductive tract infections. CID 2017; 00(00)1-9.

*f. Data collection and monitoring*

The study was implemented according to the approved protocol and study specific study operating procedures (SOPs). Data collection for the trial continued until all malaria safety monitoring had been completed at delivery and after infant follow-up. Questionnaire data was entered directly into an electronic Case Report Form (CRF) on MACRO (InferMed, UK), Good Clinical Practice (GCP) compliant software for clinical trials. Weekly follow-up visits were recorded on electronic questionnaires using Personal Digital Assistant (PDA) handheld devices with in-built consistency checks. PDA data was uploaded weekly onto Macro. An external independent trial monitor from the Institute of Tropical Medicine in Antwerp assessed SOP adherence and reported to the Sponsor on GCP compliance and trial conduct on three occasions. An internal monitor not involved in this trial, verified on a continuous basis that the rights and well-being of human subjects were protected and that the trial was conducted in compliance with the approved protocol on a six monthly basis. A Data Safety Monitoring Board met four times during the course of the trial.
