# Supplementary material for: Effects of long-term weekly iron and folic acid supplementation on lower genital tract infection – a double blind, randomised controlled trial in Burkina Faso
Source: BMC Med. 2017 Nov 23;15:206. doi: 10.1186/s12916-017-0967-5 (PMC5700548; doi:10.1186/s12916-017-0967-5)
Supplement: Supplementary file 2 — Detailed methods for microbiota and T. vaginalis qPCR profiling. (DOCX 13 kb) [file 12916_2017_967_MOESM2_ESM.docx]

**Additional File 2**

**Microbiota/T.vaginalis qPCR methods**

PCR used 1x Accuprime Pfx Supermix, 0.5 µM of each primer and 1 µl template DNA under the following conditions: 95⁰C 2 minutes, 30 cycles 95⁰C 20s, 55⁰C 15s, 72⁰C 5 minutes with a final extension 72⁰C 10 minutes. A positive and negative control sample were included in each plate and carried through to sequencing. PCR products were normalised using SequalPrep™ Normalization kit (Invitrogen) following manufacturer instructions and combined into four pools. Each pool was quantified using fragment size determined by BioAnalyzer (Agilent Technologies) and concentration by Kappa qPCR (Kappa Biosystems). Pools were combined in equimolar amounts to create a single library then denatured using 0.2N NaOH for 5 minutes followed by 2 minute incubation at 96⁰C. The library was diluted to a final concentration of 3.5 pM and supplemented with 5% PhiX and loaded onto a MiSeq V2 2x250 cartridge. Raw fastq files were processed using Mothur (v1.35.1) (Kozich et al. 2013). Sequences were aligned to the Silva database and chimeric sequences were detected by Chimera.uchime and removed from downstream analysis. A cut-off of 70 was applied to assign sequences to the RDP trainset9_032012. The get.oturep command was used in Mothur to retrieve representative sequences. Sequences were assigned putative species level IDs using the RDP sequence match function. Dominant operational taxonomic units (OTUs) in the PCR and kit negative controls belonged to *Escherichia*, *Pseudomonas*, *Enterococci* and *Staphylococci genera*, which were also present in samples. To investigate if these OTUs belonged to viable bacteria those with these predominant OTUs were cultured (n=40). Samples were diluted in phosphate buffered saline and cultured aerobically and anaerobically on Columbia blood and Brain Heart Infusion agar. Isolates were sub-cultured on appropriate media and identified by matrix assisted laser desorption ionization time-of-flight mass spectrometry (MALDI-TOF MS). Escherichia and Pseudomonas were not isolated from any samples and excluded from analyses. Each sample was rarefied to 5000 reads for final analysis.

Analysis and visualization of microbiota communities was conducted in R, utilizing the phyloseq package to import data and calculate alpha- and beta-diversity metrics. Significance of categorical variables used a non-parametric Mann-Whitney test for two category comparisons, or the Kruskal-Wallis test for comparison of ≥ three categories. P values were adjusted for multiple comparisons with the FDR algorithm.

An R script implemented for vaginal microbiota analysis [R. foundation] was employed for partitioning around medoids (PAM) to cluster samples into community state types (CSTs). Bray-Curtis was used to calculate distance between samples and this was denoised by extraction of the most significant Principal Coordinates Analysis (PCoA) eigenvectors before applying the PAM algorithm. Gap statistics were used to determine the number of clusters based on the elbow method, selecting the number of clusters at the point where the marginal gain in explained variance begins to plateau.

*References*

Kozich JJ, Westcott SL, Baxter NT, Highlander SK, Schloss PD. Development of a dual-index sequencing strategy and cultivation pipeline for analyzing amplicon sequence data in the MiSeq Illumina sequencing platform AEM 2013; 79: 5112-20.

R: A language and environment for statistical computing. R Foundation for Statistical Computing, Vienna, Austria. R Core Team, 2016. URL <https://www.R-project.org/>.

McMurdle PJ & Holmes S. Phyloseq: an R package for reproducible interactive analysis and graphics of microbiome census data. PLoS One 2013; 8:e61217.

DiGiulio DB, Callahan BJ, McMurdie PJ et al. Temporal and spatial variation of the human microbiota during pregnancy. PNAS 2015; 112: 11060-65.
