# Supplementary material for: Effects of long-term weekly iron and folic acid supplementation on lower genital tract infection – a double blind, randomised controlled trial in Burkina Faso
Source: BMC Med. 2017 Nov 23;15:206. doi: 10.1186/s12916-017-0967-5 (PMC5700548; doi:10.1186/s12916-017-0967-5)
Supplement: Supplementary file 3 — Tables not shown in Results. (DOCX 49 kb) [file 12916_2017_967_MOESM3_ESM.docx]

**Additional File 3**

**Data not shown in main paper**

a. For each cohort, approximately one third did not attend their final assessment. Table A1 shows that participants lost from the pregnancy cohort were not very different from those who attended ANC1. For FIN those who did not attend were slightly older, less well educated, more likely to be menarcheal and to be having sex at baseline. Reasons for loss to follow-up were primarily out-migration for work, or with their husband’s migration and some women miscarried before their first antenatal visit.

b. Tables A2 and A5 are in reference to Tables 2 and 6 in the paper which presented infection markers and CST states in iron deficient versus replete women using pre-specified thresholds for adjusted ferritin and sTfR/log ferritin: ferritin < 15 µg/l if C-reactive protein (CRP) < 10 or, ferritin < 70 µg/l if CRP ≥ 10 µg/ml; and ratio of sTfR µg/ml to log10 ferritin >5.6. The trial protocol also specified iron deficiency as ferritin < 15 µg/l if C-reactive protein (CRP) < 10 µg/ml or, ferritin < 30 µg/l if CRP ≥ 10 µg/ml. Tables A2 and A5 below reproduce the relevant portions of Table 2 and Table 6 showing infection outcomes and using the alternative lower ferritin threshold. Prevalence of vaginal discharge remained significantly lower in iron replete women (P=0.013), and normal vaginal flora remained significantly less frequent in iron replete women (P=0.028) (Table A2). CST frequencies did not differ significantly from earlier results using the lower ferritin threshold (Table A5). We conclude that using a lower iron deficiency ferritin threshold did not alter infection outcomes.

c. Table A3 shows relative risks and associated P-values for the five biomarkers based on log-transformed values and normalised by the standard deviation of the biomarker so that RR refer to risks associated with a 1SD change in the biomarker. The regression model adjusts for MUAC, and unadjusted values for MUAC and BMI are shown for in the first two rows. Biomarker P-values are adjusted for multiple testing on a per-infection basis using the FDR method. N=1673 (Reference 26 main paper, Benjamini Y and Hochberg Y).

d. Table A4 shows that there were no significant associations between infection category and receipt of antibiotics in the pregnant cohort. Incidence ratios for antibiotic receipt were, however, higher for gastrointestinal, respiratory and miscellaneous infections.

**Table A1 Characteristics of pregnant and non-pregnant women not attending their ANC1 or FIN assessment and therefore included or excluded from the analysis datasets**

|  | **Pregnant Cohort** | | | **Non-pregnant Cohort ^a^** | | |
| --- | --- | --- | --- | --- | --- | --- |
| **Variable** | N ^b^ | Included | Excluded | N ^b^ | Included | Excluded |
|  |  | N=315 | N=163 |  | N=876 | N=673 |
| **Socio-demographic,** n (%) | |  |  |  |  |  |
| Age, years, mean (SD) | 478 | 17.1 (1.8) | 17.1 (1.5) | 1549 | 16.5 (1.7) | 16.9 (1.8) |
| Ethnic Group | |  |  |  |  |  |
| Mossi | 478 | 309 (98.1) | 155 (95.1) | 1548 | 855 (97.7) | 632 (93.9) |
| Other | 478 | 6 (1.9) | 8 (4.9) | 1548 | 20 (2.3) | 41 (6.1) |
| Marital Status | |  |  |  |  |  |
| Married | 478 | 29 (9.2) | 8 (4.9) | 1544 | 15 (1.7) | 20 (3.0) |
| Never married | 478 | 286 (90.8) | 154 (94.5) | 1544 | 859 (98.3) | 649 (96.9) |
| Previously married | 478 | 0 (0) | 1 (0.6) | 1544 | 0 (0) | 1 (0.1) |
| Occupation^c^ | |  |  |  |  |  |
| Student | 478 | 81 (25.7) | 30 (18.4) | 1549 | 374 (42.7) | 155 (23.0) |
| Trading | 478 | 14 (4.4) | 6 (3.7) | 1549 | 21 (2.4) | 24 (3.6) |
| Domestic labour | 478 | 177 (56.2) | 97 (59.5) | 1549 | 399 (45.5) | 409 (60.8) |
| Farming | 478 | 138 (43.8) | 93 (57.1) | 1549 | 279 (31.8) | 280 (41.6) |
| Other | 478 | 4 (1.3) | 2 (1.2) | 1549 | 4 (0.5) | 1 (0.1) |
| Education | |  |  |  |  |  |
| No Schooling | 477 | 204 (65.0) | 114 (69.9) | 1544 | 441 (50.5) | 456 (68) |
| Primary | 478 | 60 (19.0) | 32 (19.6) | 1549 | 216 (24.7) | 121 (18) |
| Lower Secondary | 477 | 46 (14.6) | 16 (9.8) | 1544 | 212 (24.3) | 85 (12.7) |
| Higher Secondary | 477 | 4 (1.3) | 1 (0.6) | 1544 | 4 (0.5) | 9 (1.3) |
| Literate | 473 | 92 (29.6) | 38 (23.5) | 1530 | 401 (46.5) | 174 (26.1) |
| **Reproductive history and infection markers**, n (%) | | | |  |  |  |
| Menarcheal | 478 | 294 (93.3) | 150 (92.0) | 1549 | 756 (86.3) | 528 (78.5) |
| Menarche age, mean (SD) | 443 | 14.9 (1.1) | 14.9 (0.9) | 1279 | 15 (1.1) | 15.1 (1.0) |
| Ever had sex | 478 | 119 (37.8) | 61 (37.4) | 1548 | 135 (15.4) | 188 (27.9) |
| Uses Contraception | 478 | 69 (21.9) | 36 (22.1) | 1549 | 83 (9.5) | 109 (16.2) |
| Uses Condoms | 478 | 67 (21.3) | 36 (22.1) | 1549 | 83 (9.5) | 101 (15) |
| Nugent 7-10 | 391 | 32 (12.3) | 24 (18.3) | 1247 | 78 (11) | 70 (13) |
| Nugent 4-6 | 391 | 23 (8.8) | 5 (3.8) | 1247 | 71 (10) | 44 (8.2) |
| Nugent 0-3 | 391 | 205 (78.8) | 102 (77.9) | 1247 | 559 (79) | 425 (78.8) |
| Vaginal discharge | 478 | 5 (1.6) | 2 (1.2) | 1549 | 13 (1.5) | 12 (1.8) |
| Vaginal pH ≥4.5 | 394 | 124 (47.1) | 79 (60.3) | 1280 | 376 (51.9) | 269 (48.5) |
| **Nutrition and iron biomarkers**, n (%) | | |  |  |  |  |
| Drinks alcohol | 478 | 0 (0) | 163 (100) | 1549 | 821 (93.7) | 671 (99.7) |
| BMI, kg/m^2^, mean (SD) | 478 | 20.1 (1.9) | 20.5 (2.0) | 1549 | 19.5 (2.0) | 19.8 (2.4) |
| MUAC, cm, mean (SD) | 478 | 24 (1.9) | 24.5 (2.1) | 1549 | 23.5 (2.0) | 23.7 (2.4) |
| Iron deficient (adj ferr) | 467 | 35 (11.4) | 16 (10.0) | 1528 | 113 (13.1) | 71 (10.7) |
| Iron deficient  (ratio sTfR/log ferritin) | 470 | 74 (24.0) | 33 (20.4) | 1524 | 186 (21.5) | 144 (21.8) |
| **Treatment** | |  |  |  |  |  |
| Allocated to Iron, n (%) | 478 | 163 (51.7) | 95 (58.3) | 1549 | 426 (48.6) | 340 (50.5) |
| DOT ^d^, mean (SD) | 472 | 74.2 (24.1) | 64.2 (29.7) | 1549 | 78.9 (17.8) | 59.3 (32.4) |

^a^ Includes only participants menarcheal for at least six months before FIN

^b^ Total responders

^c^  More than one response allowed

^d^ Number of directly observed treatments as a percentage of the number of weeks from enrolment to assessment

**Table A2 Baseline host iron status in relation to infection markers using the alternative lower iron deficiency threshold * (n=1673, excluding 281 non-menarcheal)**

|  |  |  |  | | **Unadjusted** | | **Adjusted ^a^** | | | |
| --- | --- | --- | --- | --- | --- | --- | --- | --- | --- | --- |
| **Infection marker** | **N** | **Replete (%)** | **Deficient (%)** | | **RR (95%CI)** | **P ^b^** | **N ^c^** | **RR (95%CI)** | | **P ^b^** |
| BV ^d^ | 1347 |  |  |  | | 0.175 | 1326 |  |  |  |
| Nugent 7-10^e^ | 1347 | 144/1172 (12.3) | 13/154 (8.4) | 1.46 (0.85-2.50) | | 0.186 | 1326 | 1.53 (0.89-2.64) |  |  |
| Nugent 4-6 ^e^ | 1347 | 109/1172 (9.3) | 10/154 (6.5) | | 1.43 (0.77-2.68) | 0.295 | 1326 | 1.41 (0.75-2.64) | | 0.284 |
| Nugent 0-3 ^e^ | 1347 | 919/1172 (78.4) | 131/154 (85.1) | | 0.92 (0.86-0.99) | 0.058 | 1326 | 0.92 (0.86-0.99) | | 0.028 |
| Vaginal discharge | 1673 | 21/1452 (1.4) | 8/192 (4.2) | | 0.35 (0.16-0.77) | 0.015 | 1644 | 0.36 (0.16-0.81) | | 0.013 |
| pH>=4.5 | 1371 | 621/1187 (52.3) | 73/162 (45.1) | | 1.16 (0.97-1.39) | 0.094 | 1349 | 1.16 (0.97-1.39) | | 0.107 |

- Ferritin < 15 µg/l if C-reactive protein (CRP) < 10 µg/ml or, ferritin < 30 µg/l if CRP ≥ 10 µg/ml

^a^ Adjusted for MUAC

^b^ Fishers Exact Test

^c^ Number of observations in adjusted analysis

^d^ Global test (Fishers/ordinal regression) for BV as 3 category outcome

^e^ Each group compared to both other groups

**Table A3** **Association between nutritional and iron biomarkers with infection markers at baseline**

| **Variable** | **Bacterial vaginosis** | | **Vaginal pH≥4.5** | | **Vaginal Discharge** | |
| --- | --- | --- | --- | --- | --- | --- |
|  | **RR (95% CI)** | **P** | **RR (95% CI)** | **P** | **RR (95% CI)** | **P** |
| MUAC cm ^a^ | 1.23 (1.08-1.41) | 0.003 | 0.99 (0.94-1.04) | 0.63 | 1.26 (0.90-1.75) | 0.18 |
| BMI kg/m^2^ ^b^ | 1.23 (1.08-1.40) | 0.002 | 0.99 (0.94-1.05) | 0.76 | 1.02 (0.72-1.45) | 0.90 |
| Ferritin µg/l | 1.10 (0.95-1.27) | 0.26 | 1.08 (1.03-1.14) | 0.015 | 0.69 (0.50-0.96) | 0.068 |
| sTfR µg/ml ^c^ | 0.91 (0.81-1.01) | 0.18 | 0.98 (0.94-1.03) | 0.39 | 0.94 (0.68-1.31) | 0.72 |
| sTfR/log_10_ ferritin ratio | 0.89 (0.78-1.01) | 0.18 | 0.96 (0.92-1.01) | 0.14 | 1.19 (0.84-1.68) | 0.41 |
| CRP mg/l ^d^ | 1.01 (0.87-1.17) | 0.90 | 0.97 (0.92-1.02) | 0.25 | 0.67 (0.47-0.95) | 0.068 |

^a^ Mid-upper-arm-circumference

^b^  Body mass index

^c^ Serum transferrin receptor

^d^ C-reactive protein

**Table A4 Mean number of Health Centre antibiotic treatments in pregnant cohort by infection diagnosis and trial arm from enrolment to ANC1**

| **Infection**  **category** | **Iron** | **Control** | **Iron**  **per 50 visits** n=6907 | **Control**  **per 50 visits**  n=6235 | **Incidence Ratio ^a^** | **P value** | Pfdr ^b^ |
| --- | --- | --- | --- | --- | --- | --- | --- |
| Malaria ^c^ | 8 | 9 | 0.058 | 0.072 | 0.80 ( 0.31 - 2.09 ) | 0.651 | 0.837 |
| Respiratory | 26 | 14 | 0.188 | 0.112 | 1.68 ( 0.87 - 3.22 ) | 0.119 | 0.448 |
| Local ^d^ | 10 | 11 | 0.072 | 0.088 | 0.82 ( 0.35 - 1.94 ) | 0.651 | 0.837 |
| Gastrointestinal ^e^ | 15 | 7 | 0.109 | 0.056 | 1.93 ( 0.79 - 4.76 ) | 0.149 | 0.448 |
| Urinary Tract ^f^ | 2 | 4 | 0.014 | 0.032 | 0.45 ( 0.08 - 2.48 ) | 0.358 | 0.645 |
| STI ^g^ | 9 | 0 | 0.065 | 0 | NA | 0.992 | 0.995 |
| Genital ^h^ | 14 | 21 | 0.101 | 0.168 | 0.60 ( 0.31 - 1.19 ) | 0.141 | 0.448 |
| Dental | 0 | 2 | 0 | 0.016 | NA | 0.995 | 0.995 |
| Miscellaneous ^i^ | 7 | 3 | 0.051 | 0.024 | 2.11 ( 0.54 - 8.19 ) | 0.280 | 0.631 |

1. Incidence ratio was computed using Poisson regression with number of visits as exposure period
2. P value adjusted for multiple testing using false discovery rate method
3. Antibiotics in addition to anti-malarial treatment
4. Non-enteric and non-respiratory, includes ear, skin, ophthalmic, abscess, wounds, local trauma, sinusitis, mastoiditis, keloid, scalp ringworm
5. Dysentry, typhoid, enteritis, intestinal parasitosis, gastric ulcer, gastroenteritis, diarrhea and abdominal pain, vomiting and abdominal pain, amoebiasis, colopathy, sub-occlusion
6. Cystitis, dysuria
7. Includes syphilis, trichomoniasis
8. Upper or lower genital infection
9. Itching, colic, headache, generalized or localized pain, fever, vomiting only, anaemia, anxiety, neuralgia, spasms, urticarial, plus non-infectious specific diagnoses: cancers, fertility problems, angina, self-medication, allergy, burns, renal stones, contraception, thyroid and cardiac diseases, epilepsy, filariasis, foreign body, venomous bites, fractures, migraine, HIV, varicella, mumps, prophylactic and non-classifiable: includes 3 uncertain descriptors, or absent statement

**Table A5 Community State Type distribution in pregnant and non-pregnant (menarcheal) cohorts by iron deficiency and infection status using the alternative lower iron deficiency threshold ***

|  | **Visit** | **Iron deficiency** | **n** | **CST I**  **n (%)** | **CST III**  **n (%)** | **CST IV**  **n (%)** | **P ^a^** |
| --- | --- | --- | --- | --- | --- | --- | --- |
|  | ANC1 | No | 256 | 121 (47.3) | 78 (30.5) | 57 (22.3) | 0.431 |
|  |  | Yes | 20 | 7 (35.0) | 6 (30.0) | 7 (35.0) |  |
|  | FIN ^b^ | No | 623 | 237 (38.0) | 141 (22.6) | 245 (39.3) | 0.382 |
|  |  | Yes | 62 | 20 (32.3) | 12 (19.4) | 30 (48.4) |  |

- Ferritin < 15 µg/l if C-reactive protein (CRP) < 10 µg/ml or, ferritin < 30 µg/l if CRP ≥ 10 µg/ml

^a^ P value for association between status and CST from a multinomial regression model adjusting for antibiotic use in the three months prior to assessment

^b^ Menarcheal women only
