# Supplementary material for: Effects of long-term weekly iron and folic acid supplementation on lower genital tract infection – a double blind, randomised controlled trial in Burkina Faso
Source: BMC Med. 2017 Nov 23;15:206. doi: 10.1186/s12916-017-0967-5 (PMC5700548; doi:10.1186/s12916-017-0967-5)
Supplement: Supplementary file 4 — (a) Legend to Additional figures; (b) CRP sensitivity analysis and iron deficiency; (c) Figure Shannon Diversity (microbiota results). (ZIP 116 kb) [file 12916_2017_967_MOESM4_ESM.zip › Additional File 4a Figure legendsR3.docx]

**Additional File 4a**

**FIGURE LEGENDS**

**File 4b Iron deficiency prevalence by C-reactive protein concentration cut-offs**

We include a plot of the proportion iron deficient in the two trial arms at baseline (blue – control, red – iron, with 95%CI) where the CRP cut-off definition is allowed to vary by around a factor of two. This demonstrates that while the absolute deficiency levels vary with alternative CRP values if alternative definitions are used, the trends and arm differences remain similar. The vertical line corresponds to the CRP definition applied in the analysis (< 10 µg/ml). Iron deficiency cut-offs are ferritin <15 µg/l for low CRP, and ≤ 70 µg/l for high CRP.

**File 4c Boxplot showing species richness and Shannon diversity for pregnant (ANC1) and non-pregnant (FIN)**.

Results are shown by a) trial arm and b) iron deficiency using the ratio of serum transferrin receptor μg/l sTfR to log_10_ ferritin >5.6 (using sTfR of 8.3μg/ml and log_10_ ferritin of 30μg/l and CRP ≥ 10μg/ml (adjFE).
