# Supplementary figures and images for: Effects of long-term weekly iron and folic acid supplementation on lower genital tract infection – a double blind, randomised controlled trial in Burkina Faso
Source: BMC Med. 2017 Nov 23;15:206. doi: 10.1186/s12916-017-0967-5 (PMC5700548; doi:10.1186/s12916-017-0967-5)

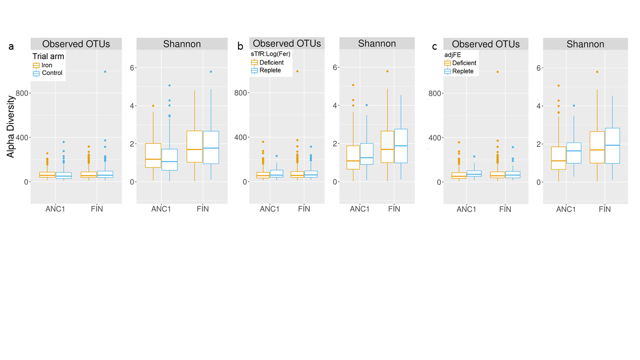

Supplement: Supplementary file 4 — (a) Legend to Additional figures; (b) CRP sensitivity analysis and iron deficiency; (c) Figure Shannon Diversity (microbiota results). (ZIP 116 kb) [file 12916_2017_967_MOESM4_ESM.zip › Additional File 4c CST frequencies Revision 3R3.tif]
